# Genome-Wide Survey of Cohesin: A Molecular Guardian of Genomic Fidelity

**DOI:** 10.1371/journal.pbio.0020291

**Published:** 2004-07-27

At a fundamental level, the continuity of life depends on cell division. Humans generate many millions of cells per second just to stay alive, with most cell types dividing and multiplying repeatedly during a lifetime. Details of cell division vary from cell to cell and organism to organism, but certain features are universal, including what is arguably a cell's most crucial task: the faithful duplication and segregation of its genetic material.

During mitosis, a cell copies its nuclear DNA, then splits into two identical daughter cells, a process that involves moving the replicated chromosomes (called sister chromatids) toward opposite ends of the cell. After chromosomes replicate, a protein complex called cohesin binds the sister chromatids together. Cohesion helps the cell distinguish between the copies, which in turn aids proper distribution. Improper sister chromatid segregation can yield an abnormal number of chromosomes (called aneuploidy) in the daughter cells, a condition associated with cancer. During meiosis—the cell division that produces egg and sperm cells—aneuploidy causes a number of congenital disorders, including Down's syndrome.[Fig pbio-0020291-g001]

**Figure pbio-0020291-g001:**
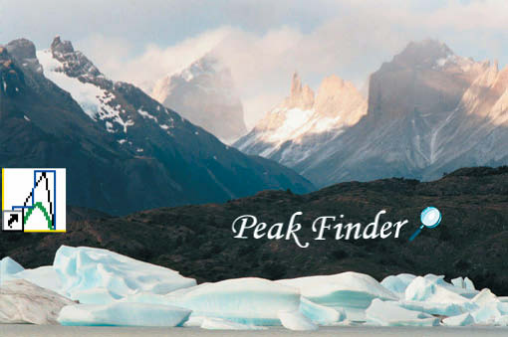
PeakFinder automates identification of peaks in ChIP data

To end up in their appropriate positions, sister chromatids must establish attachments to tentacle-like protein polymers called spindle microtubules, which emanate from spindle poles at opposite ends of a cell. Cohesion between the chromatids makes these bipolar attachments possible and keeps sister chromatids from separating after they attach to the spindle. Cohesion occurs along the length of a chromosome and is particularly strong around centromeres, the pinched region of a chromosome. Centromeres, in turn, assemble another protein complex called the kinetochore, which mediates the attachment of chromosomes to spindle microtubules; together, they guide chromosomes to their respective destinations.

Cohesin's binding locations were discovered by removing chromatin—the mass of DNA and proteins that forms chromosomes—from cells, and purifying the regions associated with cohesin. These studies looked at cohesin's binding distribution either genome-wide or at select regions of a few chromosomes. Here, two research groups use a similar approach to provide a broader picture in their analysis of cohesin binding in the budding yeast Saccharomyces cerevisiae, a favorite system for cell biologists. In the first paper, Jennifer Gerton and colleagues generated a map for the entire yeast genome of locations where cohesin binds to chromosomes during meiosis and mitosis. In the second paper, Paul Megee and colleagues found that centromeres attract large concentrations of cohesin to their flanks and that the assembly of these cohesin domains is mediated by centromere–kinetochore complexes.

Gerton's group reports that large regions surrounding centromeres have “intense” cohesin binding. These binding sites correlate with DNA base composition—DNA is composed of four chemical bases, or nucleotides, that are referred to as A, C, G, and T—showing a strong association with AT-rich regions. In meiotic chromosomes, cohesin binding sites are interspersed between the DNA double-strand breaks that initiate the exchange of genetic information characteristic of meiosis, perhaps keeping the chromatids attached without interfering with genetic recombination.

Most striking, the authors note, is the observation that cohesin binding changes according to the cell's gene transcription program. Cohesin prefers DNA that lies between active transcription zones and is unceremoniously displaced from regions where RNA transcripts are being made (a process called elongation). This suggests that elongation through a region and cohesion binding may be incompatible. These observations support previous work indicating that DNA sequences required for the replication and segregation of chromosomes must be protected from transcription to function properly. Whatever the explanation, this finding begs the question of how more complicated genomes can accommodate these two seemingly contradictory processes.

Megee's group investigated whether all yeast chromosomes have these large centromere-flanking cohesin regions and whether the centromeres and DNA sequences that surround them somehow facilitate the assembly of cohesin complexes. By removing centromeres and generating cells incapable of assembling kinetochores, the researchers show that the assembly of these cohesin regions is mediated solely by the centromere–kinetochore complex.

What's more, inserting centromeric DNA sequences in abnormal chromosomal locations produced new cohesin-assembling regions around these “neo” centromeres. The kinetochores' influence appears to stretch over tens of thousands of DNA bases and serves chromatid segregation in two crucial ways: by recruiting high levels of cohesin to centromeres' sides, which attaches chromatids to their bipolar spindles, and by attaching chromatids to microtubules, which provides their passage to the cell's opposite sides. The maintenance of genomic integrity, the authors conclude, likely relies on the coordination of these essential functions.

